# Complete Genome Sequence of *Bacillus kochii* Oregon-R-modENCODE Strain BDGP4, Isolated from *Drosophila melanogaster* Gut

**DOI:** 10.1128/genomeA.01074-17

**Published:** 2017-10-05

**Authors:** Kenneth H. Wan, Charles Yu, Soo Park, Ann S. Hammonds, Benjamin W. Booth, Susan E. Celniker

**Affiliations:** Biological Systems and Engineering Division, Lawrence Berkeley National Laboratory, Berkeley, California, USA

## Abstract

*Bacillus kochii* Oregon-R-modENCODE strain BDGP4 was isolated from the gut of *Drosophila melanogaster* for functional host microbial interaction studies. The complete genome comprised a single chromosomal circle of 4,557,232 bp with a G+C content of 37% and a single plasmid of 137,143 bp.

## GENOME ANNOUNCEMENT

The *Bacillus* genus represents one of the oldest, most ubiquitous, and molecularly well-characterized bacterial clades. *Bacillus kochii*, first described in 2012, is aerobic, motile, and catalase positive (1). This report describes the first complete sequence of *B. kochii* Oregon-R-modENCODE strain BDGP4, associated with a sequenced *Drosophila melanogaster* host facilitating mechanistic studies of host–microbiome interactions.

*Bacillus kochii* Oregon-R-modENCODE strain BDGP4 was isolated from a fecal swab. Bacteria were streaked onto nutrient broth agar (BD catalog no. 213000) plates; single colonies were amplified in culture, and an aliquot was used for 16S V1 and V4 PCR (2) and sequence identification (3). DNA for sequencing was isolated (4), and whole-genome DNA sequencing was performed by the National Center for Genome Resources (NCGR, Santa Fe, New Mexico, USA) using Pacific Biosciences (PacBio, Menlo Park, CA, USA) long-read sequencing on the RS II instrument (5). A single-molecule real-time (SMRT) cell library was constructed with 5 to 10 µg of input DNA using the PacBio 20-kb protocol. The library was loaded onto one SMRT cell and sequenced using P6 polymerase and C4 chemistry with 6-h movie times. Sequencing yielded a total of 20,658 reads with a filtered mean read length of 13,027 bp, totaling 269,115,728 bp (>50-fold coverage). The files generated by the PacBio instrument were used for a *de novo* assembly constructed using the hierarchical genome assembly process (HGAP2) protocol from SMRT Analysis version 2.0 (6, 7). This protocol relies on BLASR for alignment (8), the Celera assembler for assembly (7), and Quiver for consensus polishing (6). The final contigs were manually trimmed and reviewed to produce a single circular chromosome and a single plasmid. Annotations of protein-encoding open reading frames and noncoding RNAs (ncRNAs) were predicted using the RAST tool (9) and the GenBank annotation pipeline (10).

The chromosomal genome annotation predicts 4,296 protein-coding genes, 10 rRNA operons, 107 tRNAs, and 5 ncRNAs. Of the 4,296 protein-coding genes, 72 are contained within candidate prophages. Our strain contains a single near-complete cryptic prophage and one partial copy (https://omictools.com/phage-finder-tool). The cryptic prophage is 39,935 bp and the partial copy is 13,008 bp. They constitute 1.16% of the genome. The 39-kb cryptic prophage contains genes encoding the large and small terminase subunit proteins but lacks an essential portal protein that is contained within the partial copy (11). In addition, the genome contains a single plasmid pBkBDGP4A (137,143 bp, 34% G+C) encoding 131 predicted genes, of which three are predicted conjugal transfer proteins and one is predicted to encode a MerR protein, known to respond to environmental stimuli, such as oxidative stress, heavy metals, or antibiotics (12). The plasmid is also predicted to encode three germination proteins—GerKA, GerKB, and GerKC—that may affect the sporulation and germination processes of the host.

### Accession number(s).

The complete chromosome and plasmid sequences of *Bacillus kochii* Oregon-R-modENCODE strain BDGP4 have been deposited in GenBank under accession numbers CP022983 (chromosome) and CP022984 (plasmid).
